# The Hostile Hospital: Exploring Hospitality, Violence, and the Doctor-Patient Relationship

**DOI:** 10.2147/JMDH.S389728

**Published:** 2024-08-14

**Authors:** Stephen R Milford, Giorgia Lorenzini

**Affiliations:** 1 North-West University, Potchefstroom, South Africa & Institute for Biomedical Ethics, Basel University, Basel, Switzerland; 2 Institute for Biomedical Ethics, Basel University, Basel, Switzerland

**Keywords:** doctor-patient relationship, paternalism, hostility in hospitals, Levinas, Derrida

## Abstract

Lévinas and Derrida speak of the ontological context of human relationships in the context of the absolute priority of the Other and the unconditional law of hospitality. This has direct implications for doctor-patient relationships in the context of health care. This paper explores these philosophical and practical implications in light of a paradox that exists in all hospitality: that hostility is inevitably intertwined with hospitality. The paper explores three ways hostility can present in doctor-patient relationships: in physical violence, through paternalism, and through the violence of categorisation. While acknowledging the paradox, and the complexity of solutions, the paper considers ways to minimize this hostility. In so doing, it encourages HCPs to overcome whatever is possible so as to do the impossible: provide unconditional hospitality.

## Introduction

Health care professionals (HCP), including doctors, nurses, care home assistants etc., form unique relationships with patients. These interactions are more than impersonal transactions between a service provider (care-givers) and service receiver (care-receivers).^1^ The HCP is the person we seek out when we are struggling, the hospital the place we run to when we are vulnerable. In our suffering, we seek someone to help us, someone who can care for us, and make us whole again.^2^ On the other hand, HCPs often choose the health care profession because they have a desire to help those who are suffering; they often have a commitment to open their hearts, and to make space for us. In other words: to offer us hospitality. Notwithstanding such noble ideals, this hospitality does not come without risks. As we will show here, it is even tinged with violence, a kind of violence that is hostile to our very being.

In our project here we wish to draw attention to an inherent paradox: Hospitality and hostility are inseparably intertwined. We will begin our discussions by sketching the roots of this paradox and move on to consider three possible forms this hostility may take in the context of health care hospitality. To do this we will briefly outline one way of understanding HCP-patient relationships through the lens of two philosophers: Lévinas and Derrida. These eminent philosophers have done much to explore the concepts of the absolute priority of the Other and the unconditional law of hospitality that each person owes another. This has implications for HCP-patient relationships everywhere. We are not the first to link the philosophical concept of hospitality to the medical profession.^3^,^4^ However, what has gone unrecognised is the inevitable hostility that accompanies all hospitality. This is a kind of violence that is at once inevitable, and yet must be overcome. This article will explore this inevitability, highlighting to medical professionals the inherent paradox of offering hospitality to patients while maintaining necessary limits.

Before we begin, a word of caution; we discuss in this paper the ideals to which the medical profession strives. These ideals are high but also impossible to achieve. We acknowledge that the context of many care institutes necessitates practices that may go counter to these ideal, but are nevertheless necessary in the “real world.” Resources (time, money, human, physical space etc.) are often limited, and this requires tough decisions be made. Our project is not a critique of the work done by HCPs. Our project is an encouragement to contemplate this paradox, not just to find solutions (which are often highly complex, impractical, and beyond the scope of this paper), but also to gain a deeper understanding of the existential nature of the inter-human activity that is health care.

## The Dwelling Self

Lévinas contends that the Self (you, I, they) exists in a world of alien objects and subjects: elements that are other to itself.^5^ At the mercy of these elements, and driven by a concern for its own security and future stability, the Self must attempt to master the world around it.^6^ To do this it must establish a safe site: a habitation or home. The process of habitation takes the form of self-recollection, whereby the Self collects and recollects its innate capacity for subjective thought and action.^5^,^6^ Through this process of recollection, the Self establishes a shelter by which it finds refuge from the alien elements that surround it. This self-constructed site is the Self’s home. In this way the Self avoids being “cast forth and forsaken in the world.”^5^ It is safe in a private domain, a site to which it can retire and recollect itself when needed. It is only because of this home that the Self may dwell in the world.

The process of habitation requires a level of mastery of the elemental world.^5^ The Self must take control of elements both within and without. It must understand itself in relation to these elements so as to navigate them. There are many tools by which the Self may accomplish this. For example, using the tool of categorisation, the Self can order and structure elements both within and without. Placing components of the world into classes and groups creates structures that help the Self understand relationships between these elements, classes, and groups. This provides the Self with a framework by which it may make sense of the world, give it meaning and significance. Most importantly, it enables the Self to modify and use these structures for the benefit of itself.

Through the process of habitation and recollection the Self comes to know something of itself and also something of the world it inhabits (both objects and subjects). The home is, therefore, the necessary region from which all human endeavours originate, it is the very condition of subjective human action.^6^ Having created a home, the Self must choose its actions and responses to the exterior world. It may shut itself off, creating a self-enclosed world marked by its own Self autonomy, divorced from the exterior realm of alterity. To Lévinas this response “evinces the absolute truth, the radicalism, of separation”^5^ that ultimately creates a “pagan shrine.”^6^

Such a pagan shrine may be legitimate in a world of objects, yet the world is composed of other selves: strangers which Lévinas refers to as the ‘Others’. To Lévinas, the Other is not a relative alterity; it is not something that can be compared to the Self so as to compare what is like and not like the Self. To do so would simply place the Other as part of a categorizable totality that the Self may control or subsume for its own purposes. Rather:

The Other remains infinitely transcendent, infinitely foreign; his face in which his epiphany is produced and which appeals to me breaks with the world that can be common to us, whose virtualities are inscribed in our nature and developed by our existence.^5^

The Other is absolutely other, so much so that there is no comparison between the Self and the Other: “The collectivity in which I say ‘you’ or ‘we’ is not a plural of the ‘I’. I, you – these are not individuals of a common concept.”^5^

It is, of course, inevitable that the Self will come into contact with the Other. When this happens, the Self has an ethical choice to make. It may respond to the Other as an object, or perhaps a different version of itself. In so doing it may attempt to control the Other, placing itself over and above the Other, or perhaps assume the Other into one of its categories and use the Other for its own purposes. Unfortunately, this is the tendency of human beings in general. Our egocentric attitude often thinks of others as extensions of ourselves, or as objects that can be manipulated for our own advantage. To be fair, this is the basis of empathy and carries with it some advantages. Yet, as Kant warns, others are not to be treated as a means to an end, but as ends in themselves.^7^ To subsume the Other into our system is to reduce the Other to what they are not: an object without subjective thoughts or desires.^5^

On the other hand, the Self may respond with hospitality. By this, Lévinas has in mind that the Self opens up its home, invites the Other in and provides for the Other a refuge. According to Derrida,^8^ Kant understood this not as philanthropy (love for a fellow human being). It is a right the Other has simply because they share the world,^8^ and it is unavoidable that the paths of the Self and the Other will cross. In this sense it is a duty, or obligation, on the part of Self to be open and hospitable to the Other. To Lévinas, the presence of the Other, whom the Self meets face-to-face, “nude and bare” – to use Wild’s rephrasing^5^ – places upon the Self the obligation to respond to the Other with absolute priority.^4^ Before them the Self can hold nothing back but must offer up what Derrida terms “unconditional hospitality”.^8^ Derrida goes so far as to claim that “only an unconditional hospitality can give meaning and practical reason to any concept of hospitality” whatsoever.^4^

This philosophical understanding of the ontological condition of all persons has implications for HCPs and the medical profession in general. It offers a helpful way of seeing the HCP-patient relationship, and a caution about the challenges inherent in this relationship.

## Hospitals as Hotels, Doctor’s as Hosts, Patients as Guests

The heading is provocative, almost as if we are demeaning both hospital and doctor. However, this is far from our intent. On the contrary, the heading simply expresses the etymological reality.^9^,^10^ The term *hospital* has Latin roots linked to *hospitalis* – a verb derived from the noun *hospitalitas –* that includes the root *hote/*hospes (host and, contradictorily, guest). These roots speak to the condition of a guest-host relationship, and it is from these Latin roots that we get a family of words that include: hospitality, hostel, hotel, and even hospital and hospice.^10^ Through this etymological reading, we can claim that the hospital, at its heart, is a place where strangers (patients) can come to receive necessary hospitality from HCPs who act as hosts.^4^

The Stranger that presents themselves to the HCP places upon the HCP an obligation of response. As described above, the HCP may reduce the patient to the same status as other objects by which the HCP is surrounded; placing the patient under internal categories to be used for the HCP’s own purposes. For example, a doctor may categorise the patient as simply another patient with another fascinating disease to be treated in exchange for personal gain (eg a pet interest, financial gain, social status, balm for the doctors’ own conscious). Yet, as we have noted, doing so is to treat the patient as an object rather than a subjective being. This diminishing would represent a harm to the patient’s recollected Self.

Naturally HCP do not wish to treat patients as objects. Yet they often need to categorise patients as if they were objects. This is both understandable and desirable. HCPs have numerous calls on their limited resources. The workload may be intense, there are often a large number of patients and only a limited number of hours to treat them all. There are often legitimate reasons for HCPs to respond to patients as objects of a similar category. Nevertheless, the effect remains: reducing patients to simply objects, or even to the category of *patient*, is an injustice to the phenomenon of experiencing the Other – the Stranger – who comes before the HCP face-to-face, not as an alter ego, or another “self”, but in their strangeness “nude and bare.” They ask not to be subsumed into the same system created by the HCP and institutions, but to be recognised as fully and completely Other. The one over whom the HCP has no power.

Recently there has been a critique of past HCP-patient relationships that were at times paternalistic.^11–13^ Within these paradigms – under the guise of “for the patient”s own good’ – well-meaning doctors, nurses and other HCPs rode rough-shot over patient autonomy, dictating treatment plans to patient. Over the last few decades much work has been done to affirm core principles in biomedical ethics including the patient’s right to autonomy and to the preference for shared decision-making processes.^14–17^ That is to say, the patient is to be treated not as an object for the purposes of the HCP, but as a thinking subject who has freedom and choice over what befalls them. There is a push recently to recognise the patient’s right to unconditional hospitality, to enter the HCP’s site, and to receive refuge; compassion; respite; and care as an absolute priority.^4^ This has brought many changes to the way patients are treated by HCPs and institutions. Many of which have been incredibly positive. Yet there remains a question: While laudable and desirable as this may appear. Is it even possible for HCPs to offer unconditional hospitality?

## The Paradox: Hostility and Hospitality

Derrida argues for the unconditionality of hospitality and the absolute priority of the Other, yet even he must admit that this is impossible.^8^ Kant,^18^ who substitutes the word *hospitality* for the German *Wirtbarkeit* (which carries the implications of a host/patron who receives a guest), argues that there are conditions to hospitality. The patron, as one who remains the master of the house, never gives up their authority in their own home. Derrida^8^ understands such a conception of the host as one who maintains the truth of authority and limits the gift of hospitality offered. The Other is welcome as long as they obey the rules and, in a certain sense, submits to the authority of the host.

Consequently, unconditional hospitality is impractical. Not only are there duties for hosts and rights for guests, but there are also duties for guests and rights for hosts. We intuitively believe that hosts have a right to screen their guests, and to deny entry to those who might harm them. A guest who violates the rules of the host must surely be denied hospitality. Take for example, overstaying their welcome. By definition, hospitality is a temporary phenomenon. When a guest overstays their right of visitation they cease to be a guest, they become a resident.^9^ Consequently, while one can affirm an unconditional law of hospitality, at the same time one must also affirm the conditional laws of hospitality.^8^

These conditional laws of hospitality present to Derrida a paradox: the unconditional law of hospitality is countered by the conditional laws of hospitality. Derrida cannot see how one is able to open up their home, or to offer hospitality, without reaffirming that this house is theirs, that they are at home and that, while the Other may make themselves at home, it is on the condition that they observe the rules of the host.^8^ This “reaffirmation of mastery”^8^ creates a paradox in which hospitality is limited from the very outset. The threshold of the home is a space that beckons to be crossed, beckons the Other in, and yet at the very same time, it presents an almost unassailable obstacle to the Other:

… for there to be hospitality, there must be a door. But as soon as there is a door and windows, it means that someone has the key to them and consequently controls the conditions of hospitality.^8^

This philosophical conundrum translates into practical implications for health care contexts. The hospital is often thought of as a place of hospitality: where the hurt, broken, vulnerable Other may come to find healing, refuge, and care. Yet at the same time, hospitals and care facilities are often places filled with laws and regulations. They adopt policies of withholding non-urgent treatment from abusive patients, and HCPs are given advice on maintaining clear boundaries to limit relationships.^19^ When one enters these sites one is often faced with a barrage of rules to be followed: sit here not there, wait your turn, be quiet, turn off your phone, be a good patient etc. Naturally, such regulations are implemented for the good of everyone, but they nevertheless carry the connotation of not being fully welcomed.

To Boersma the conditional laws of hospitality speak to a type of violence that is implicit in hospitality. He struggles to see how hostility can be avoided if we restrict hospitality along Kantian lines.^20^ Afterall, surely we would violently oppose a guest who attempted to take control of the home by force? Or who puts the home in danger? Violence, in this case, would seem not only understandable, but a moral duty. On a more subdued level, we may request a guest to leave who overstays their welcome. To Boersma, this too would be a kind of violence.

At this point we should pause to address our sensibilities about the term *violence*. There are those who may want to object to Boersma’s use of this term. In common usage it has severely negative connotations, often associated with the physical. There is often a negative moral connotation to the term, whereby one enacts an evil when one resorts to violence.^20^ The idea of hospitals being places of violence seems repugnant. To be true, physical violence is not beyond the actions of healthcare facilities. Sometimes a drunk, abusive patient must be forcibly removed from the doctor’s surgery or emergency room. This is a physical act of violence against the patient with the understandable intention of protecting others: doctors, hospital staff, other patients, and maybe even the patient themselves.

Consequently, it is important to remember that violence is not always morally abhorrent, nor is it always physical.^21^ It is any act which contravenes the rights of another.^21^ It is the use of force or coercion to inflict some form of hurt or injury – even if for the greater good. The point is pertinent. We often appeal to non-physical violence as a lesser from of malice. For example, we use economic sanctions against an unfriendly country on the assumed basis that it is a morally superior act compared with a physical invasion. On one hand this is coherent. Yet while physical weapons may not be in use, suffering and harm nonetheless ensues. One may even argue that were there not to be suffering and harm, sanctions would be ineffective.

To Boersma this is simply the condition of human existence: “violence is interwoven with the fabric of the created order.”^20^ It is impossible to have a purely altruistic hospitality. Hospitality is always, on some level, narcissistic. It is impossible to affirm the Other without at the same time affirming ourselves. There are always strings attached. This applies equally to HCPs and institutions. Hospitality in the medical profession – at least within the West – is an *economy of exchange*. Hospitals and doctor’s surgeries do not provide medical care without some expectation of something in return; both physical (doctors are paid for their services), but also non-physical, for example, social status. This is especially true in professions associated with a high degree of social respectability. The doctor who opens their surgery for the Other is often greeted by society with a degree of respect and appreciation – although there is some reason to believe this is waning.^22^,^23^

Consequently, the hospitality offered by HCPs is accompanied by a tinge of in-hospitality; by a kind of violence that we cannot seem to escape from, and which takes many forms. Let us explore three practical examples.

## The Physical, Authoritarian, and Categorial Violence Presented to Patients

The first example is perhaps the easiest to define: the threat of physical violence. Patients are guests who are always under the threat of physically being restrained, evicted, and possibly even assaulted if they are do not obey the rules set by the health care institution. While at first this appears to be only natural, questions arise as to who decides when this physical violence is enacted on the patient and to what extent. It is true that patients are at times violent to HCPs, yet there is evidence of medical professionals using physical violence inappropriately.^24^,^25^ This creates a context of power-imbalance elsewhere throughout the medical professions. Patients enter medical institutions (eg doctor’s surgeries, hospitals, or care homes) not only vulnerable medically, but also vulnerable to the threat that the host may physically reject them. They have heard, have read, and have been told that other patients have been violently treated.

This threat is often blatantly obvious, although not openly stated. For example, when one is a guest of an ordinary person’s home, one is not given any indication of the possibility of physical violence. Implicitly the host may call the police if one begins to act strangely. Explicitly any such idea would be considered extremely rude and distasteful. Yet, it is common within medical facilities for the threat of violence to be openly displayed. Perhaps there is a guard at the entrance (they may even be armed), there is often a sign stating: “right of admission reserved”, and often there are numerous notices openly stating that abusive behaviour will not be tolerated. In some cases these notices will give a list of possible recursive actions which may include calling security or the authorities.

This first example of hostility has practical implications for patients, HCPs and health care institutions (HCI). The threat of violence places patients under the obligation to physically behave in a manner that they perceive is acceptable to the host. This obligation is not always easy to adhere to in the context of patient suffering. Let us consider one example (although there are others). Consider patients who suffer from mental illness, or are addicted to substances that might make them aggressive (alcohol or drugs). In the past, HCIs, and a small number of HCPs, refused to treat abusive patients. Yet we are now beginning to understand that patients are not always able to control abuse behaviour. Consequently, many countries are now implementing safeguarding practices to limit the use of physical violence, even against abusive and physically violent patients. Yet it is clear that this is not universally implemented.^26^

Derrida’s law of unconditional hospitality requires that we consider the conditional laws we implement in pursuing hospitality. As difficult as it may be, and the authors acknowledge it is often incredibly difficult, patients who are acting in ways that make providing hospitality challenging (such as being abusive) should still be offered hospitality (treatment) wherever possible. We should go to great lengths to avoid violence/abuse as much as possible from both the patient and the HCI. Nevertheless, patient violence and abuse should not prohibit necessary hospitality (treatment). It may be that HCIs need to make use of different security measures (perhaps better trained security staff who deal with patients in compassionate ways), or that HCPs are equipped to treat abusive patients in ways that affirm hospitality but do not endanger HCPs. Whatever we do, we should resist the urge to match a patient’s physical violence and abuse, with our own physical violence and abuse.

The second example of violence is well documented, that of paternalism.^11–13^ Hospitality always takes place in a location; physical and non-physical. Surgeries, hospitals, care homes etc. are often physical sites, within which clear hosts (doctors, nurses, and medical staff) retain the mastery of the dwelling. Yet, as we have alluded to above, the site may be non-physical. Medical professionals are habituated in the dwelling of the medical profession whereby they have mastery over the elements of disease, illness, and human brokenness. This mastery does not imply the ability to cure all aliments. Rather, it speaks of the ability to dwell – and often thrive – within this context.

Patients often find themselves cast adrift and without a dwelling within the elements of human illness, disease, and suffering. They do not always have the skills, knowledge, or language to anchor themselves and enact the process of recollection so as to build a shelter and place of refuge. They seek out other sites (hospitals, surgeries, care homes etc.) to find this refuge. That the HCP has a home within this foreign land (both physically and non-physically) places the patient immediately in a vulnerable position. The patient must obey the master of this dwelling (the HCP) or be jettisoned unprotected into a foreign land. This opens up the possibility of the violence of paternalism. The patient, already vulnerable, often has no choice but to obey what the host says. In many cases this may be well-meaning. The HCP is simply trying to help. In other cases it may be intentionally harmful. Nevertheless, the patient is often vulnerable to the mastery of the host. Consequently, the very context of the dwelling place of the host encourages the giving up of the guest’s (patient's) autonomy. The HCP is master, the patient simply a temporary guest. Such authoritarian contexts have long been the subject of criticism for the harm and damage done to patients.^13^

This has practical implications. Patients often do not have many of the resources such as knowledge, language, or communicative skills – which make up the Self’s dwelling – from which to question the medical professional. There is evidence to suggest that patient education, for example, can help patients to gain control over complex medical conditions.^27^,^28^ This in turn assists patients to be more independent of medical advice^29^ and ultimately leads to improved quality of life.^30^,^31^ Interestingly, better patient education does not lead to more time spent with patients^27^ and is something HCPs, and HCIs, can strive to achieve during every interaction. While there are recent moves to improve patient access to resources such as information – which could greatly assist in the process of habitation – some HCPs are resistant to more educated patients. In some cases paternalism persists and educated patients are seen as being inappropriate by non-specialised doctors who view such patients as being non-compliant.^29^ We should continue to fight against paternalism for example by emphasising that patient education is important. Providing education is one way that HCPs, and HCIs, can give patients the tools necessary to re-establish the process of habitation and, thereby, empower patients to dwell in the foreign land that is health care.

Third, there is the violence of categorisation. By necessity the medical profession uses categories to enact their profession. For example, categorising a patient as: female; 26; Caucasian; resides at such and such address; suffering from X, Y, or Z. This classification is a system of removing *the person* from the patient, focusing on the data, the diagnosis and the standard treatment. Even a diagnosis is a form of categorisation; placing the patient within a group of people who share the same illness. Here diagnosis and diagnostic codes are classifications that set rules such as what is appropriate treatment, what will be covered by insurances etc. On occasion, even stigmatised labels are used if the diagnosis is not well understood or has biased connotations (eg *fibromyalgia sufferers*). Categorisation itself is nothing controversial per se. Sacks introduced the concept of “membership categorisation” as early as 1964^32^ and since then the theory of using membership categorisations for the identification of oneself and others is well established.^33–36^ However, evidence suggest that along with certain advantages to this process, there are dangers as well.^37^ While useful and understandable from a medical perspective, in terms of patient hospitality, this represents a non-physical violence. Let us explain.

Our discussions above have demonstrated how the Other is entirely beyond our categorisation. Indeed, the very idea of categorisation is alien to the absolute priority of the Other and the law of unconditional hospitality. Categorisation is a way for the Self to understand and take hold of external elements so as to place them under its own control. It is a means to organise foreign elements and to use them for the Self’s own purposes:

… to classify him [the patient] as sick, to announce to him his death sentence… is not what I comprehend: *He is not under a category*. He is the one to whom I speak – he has only a reference to himself; he has no quiddity.^5^

What Lévinas is saying is that the very process of categorisation, even for patients, is problematic. For example, the doctor may diagnose a patient as “sick” and even provide a sub-category for their sickness – “stage 4 malignant melanoma” – yet this category risks a harm to the absolute otherness of the patient. It places the patient among other patients, as one would place an object among objects: apples to apples, oranges to oranges; diabetes to diabetes, cancer to cancer. Strictly speaking, however, a patient (who is a Self unlike any other Self), is an entity with the innate capacity for subjective thought and action. They cannot be placed in any category. To do so is an affront to their Otherness, and a breach of the unconditional law of hospitality. It is a way of controlling them (even if for their own good) and a form of objectification.

Practically, one may need to apply a category to a patient. One may need to apply the category of “HIV patient” or “ someone who has contracted dementia.” This is understandable. However, HCPs should always bear in mind the inherent hostility of such categorisation. Not all those who are HIV-positive, or living with dementia, have the same experiences. The HCP can help to alleviate this hostility by continuing to remind themselves and others – including the patient – that patients are persons and as such beyond comparison to other persons. We can encourage ourselves, HCPs, HCI and patients, to think of the categories we apply as nothing more than tools used to control the alien elements in the foreign world of health care. A category is not a designation of who or what a patient is, but an empowering tool that patients and HCPs use to exercise some form of mastery over illness and disease. In this sense the authors affirm recent moves to speak of patients with certain aliments in more affirming ways, for example: living with dementia as opposed to demented.^38^,^39^

## Conclusion: The Necessity to Do the Impossible

The hospitality offered by HCPs is inevitably tinged with hostility, both physical and non-physical. There are numerous rules to be obeyed, obligations to be met, and behaviour that must be adhered to. The sick find hosts who encourage them to *make themselves at home*, but it is always at home in a foreign land, in the home of another who is master. The door that opens for them is of a house privileged to have a door to open. It may shut to them and cast them out into an alien world at any time. Even when the door is opened for them, the host is forced to categorise them: place them within a group of supposedly similar guests and treat them the same as others. A patient’s sense of absolute alteriority is radically questioned as they are processed as just another patient.

Before lamenting the seemingly inevitability of a poisoned relationship between HCPs and patients, one would do well to bear in mind that hospitality derives from a root term that enigmatically also forms the root of its anti-thesis: *hostility*. The two coexist as opposite sides of a coin. It is not that we must give up our ideals; health care professionals must continue to affirm the absolute priority of the Other and the unconditional law of hospitality. On the contrary, we must acknowledge the paradox, learn to live with it, all the while working to overcome what is possible so as to achieve what is impossible.

All human beings are called to offer unconditional hospitality and to respect the absolute priority of the other. The HCP even more so. They have chosen a noble profession which is afforded a level of prestige precisely on the basis of this calling. It is because HCPs often demonstrate incredible self-sacrifice (sometimes even suffering abuse themselves) that they retain the esteem of the community. It is their ability to overcome the immense obstacles placed before them: limited resources; numerous calls on their time; policies and procedures that hinder their ability to extend unconditional hospitality, that we admire HCPs so much. HCPs who work within these restrictions and yet are able to counter these hostile elements so as to invite patients into their sites of refuge are to be applauded. Not because they have done something uncalled for, but because they heeded the call placed on all human beings: a call to regard the Other as a priority. While seemingly impossible, we must all observe Derrida’s words: “It is necessary to do the impossible. If there is hospitality, the impossible must be done.”^8^
